# Novel Single-Stranded DNA Virus Genomes Recovered from Chimpanzee Feces Sampled from the Mambilla Plateau in Nigeria

**DOI:** 10.1128/genomeA.01715-16

**Published:** 2017-03-02

**Authors:** Matthew Walters, Musa Bawuro, Alfred Christopher, Alexander Knight, Simona Kraberger, Daisy Stainton, Hazel Chapman, Arvind Varsani

**Affiliations:** a School of the Biological Sciences, University of Canterbury, Christchurch, New Zealand; b Nigerian Montane Forest Project, Mambilla Plateau, Taraba State, Nigeria; c Department of Microbiology, Immunology and Pathology, Colorado State University, Fort Collins, Colorado, USA; d School of the Biological Sciences, University of Queensland, Brisbane, Queensland, Australia; e Department of Clinical Laboratory Sciences, University of Cape Town, Cape Town, South Africa; f Biodesign Center for Fundamental and Applied Microbiomics, Center for Evolution and Medicine, School of Life Sciences, Arizona State University, Tempe, Arizona, USA

## Abstract

Metagenomic approaches are rapidly expanding our knowledge of the diversity of viruses. In the fecal matter of Nigerian chimpanzees we recovered three gokushovirus genomes, one circular replication-associated protein encoding single-stranded DNA virus (CRESS), and a CRESS DNA molecule.

## GENOME ANNOUNCEMENT

Through metagenomic approaches, a plethora of novel viruses have been identified. In the past five years, there has been a significant increase in the identification of single-stranded DNA (ssDNA) viruses in a wide variety of ecosystems and organisms. Animal feces have proven to harbor a rich population of viruses (1–6), and thus we sampled the feces of wild chimpanzees from a small population on the Mambilla Plateau of Nigeria to identify novel ssDNA viruses associated with chimpanzees. 

The fecal matter was processed in the same manner as described previously (1–6) and used to generate libraries for Illumina sequencing on an Illumina 2000 platform at Macrogen, Inc. (Republic of Korea). ABYSS version 1.9 was used to *de novo* assemble the paired-end reads. Viral-like sequence contigs that were >750 nucleotides (nt) in length were identified using BLASTx (7). Five contigs were identified with viral-like hits to circular ssDNA viruses. Abutting primers were designed to recover the full viral genomes using KAPA HIFI HotStart DNA polymerase (Kapa Biosystems, USA), and the resulting amplicons were cloned into the pJET2.1 vector (Thermo Fisher Scientific, USA) and Sanger-sequenced by primer-walking at Macrogen, Inc.

Three of the molecules (4,851, 5,016, and 5,184 nt) are most closely related, based on their capsid protein (CP) sequences, to members of the subfamily *Gokushovirinae* in the* Microviridae* family, and we refer to these as Chimpanzee feces–associated microphages (CfaMPs) 1 to 3. Members of *Gokushovirinae* are known to infect obligate intracellular parasites such as *Chlamydia* spp., *Bdellovibrio* spp., and *Spiroplasma* spp. (8). Diverse microvirus-like sequences have been detected in a variety of samples, including marine environments, soil, lakes, peatlands, dragonflies, humans, turkey, methane seep sediments, and bats (9–19). The genome organizations of CfaMP-1 to CfaMP-3 are similar to those of other members of the *Gokushovirinae* family. CfaMP-1 and CfaMP-3 are most closely related, sharing ~82% genome-wide pairwise nucleotide identity, whereas CfaMP-2 shares ~60 to 61% identity with CfaMP-1 and CfaMP-3. The major CP amino acid sequences of CfaMP-1 and CfaMP-3 share 81% identity with each other and 56% identity with the CP-like sequence in the bacterium *Chlamydia trachomatis* (CRH84958) and the Eel River Basin pequenoviruses (KP087936 to KP087957) from cold methane seep sediment (18). The major CP amino acid sequences of CfaMP-2 share 69% identity with isolates from an uncultured bacterium (CDL65862) recovered from a *Rattus norvegicus* isolate (20) and 47% with that of Eel River Basin pequenoviruses.

A circular replication-associated protein (Rep) encoding single-stranded (CRESS) DNA virus (3,811 nt) was also identified, which we refer to as Chimpanzee feces–associated circular DNA virus 1 (CfaV-1). The Rep of CfaV-1 is most closely related (68%) to that of rodent stool–associated circular genome virus (RodSV; JF755404) (21), whereas its putative CP shares 30% identity (58% coverage) with sequences of *Entamoeba histolytica* (EMD46443). A 2,356-nt CRESS DNA molecule was also identified that has a Rep that shares 55% and 51% amino acid identities to those of CfaV-1 and RodSV, respectively.

Diverse ssDNA viruses have been identified in a number of environmental samples, with those identified here further expanding our databases and knowledge of these ssDNA viruses.

### Accession number(s).

The complete genome sequences of the CfaMP-1, CfaMP-2, and CfaMP-3 microphages have been deposited in GenBank under the accession numbers KR704913, KR704914, and KR704915, respectively. The genome sequences of CfaV-1 and the CRESS DNA molecule were deposited in GenBank under the accession numbers KR704912 and KR704911, respectively.
